# Hyperthyroidism and Risk for Bipolar Disorders: A Nationwide Population-Based Study

**DOI:** 10.1371/journal.pone.0073057

**Published:** 2013-08-30

**Authors:** Li-Yu Hu, Cheng-Che Shen, Yu-Wen Hu, Mu-Hong Chen, Chia-Fen Tsai, Huey-Ling Chiang, Chiu-Mei Yeh, Wei-Shu Wang, Pan-Ming Chen, Tsung-Ming Hu, Tzeng-Ji Chen, Tung-Ping Su, Chia-Jen Liu

**Affiliations:** 1 Department of Psychiatry, Yuli Veterans Hospital, Hualien, Taiwan; 2 Department of Psychiatry, Chiayi Branch, Taichung Veterans General Hospital, Chiayi, Taiwan; 3 School of Medicine, National Yang-Ming University, Taipei, Taiwan; 4 Cancer Center, Taipei Veterans General Hospital, Taipei, Taiwan; 5 Department of Psychiatry, Taipei Veterans General Hospital, Taipei, Taiwan; 6 Department of Psychiatry, Far Eastern Memorial Hospital, New Taipei City, Taiwan; 7 Department of Family Medicine, Taipei Veterans General Hospital, Taipei, Taiwan; 8 Department of Internal Medicine, National Yang-Ming University Hospital, Yilan, Taiwan; 9 Department of Psychiatry, Su-Ao and Yuanshan Branch, Taipei Veterans General Hospital, Yilan, Taiwan; 10 Division of Hematology and Oncology, Department of Medicine, Taipei Veterans General Hospital, Taipei, Taiwan; 11 Institute of Public Health, National Yang-Ming University, Taipei, Taiwan; RIKEN Brain Science Institution, Japan

## Abstract

**Background:**

Thyroid disorders have long been associated with psychiatric illness, often with symptoms suggestive of mood disorders. The most common clinical features associated with hyperthyroidism are anxiety and depression. The risk of bipolar disorders, especially bipolar mania, among patients with thyroid disorders has not been well characterized.

**Objective:**

We explored the relationship of hyperthyroidism and the subsequent development of bipolar disorders, and examined the risk factors for bipolar disorders in patients with hyperthyroidism.

**Methods:**

We identified patients who were diagnosed with hyperthyroidism between 2000 and 2010 in the Taiwan National Health Insurance Research Database. A comparison cohort without hyperthyroidism was matched based on age, sex, and comorbidities. The occurrence of bipolar disorders was evaluated in both cohorts based on diagnosis and the use of mood stabilizer drugs.

**Results:**

The hyperthyroidism cohort consisted of 21, 574 patients, and the comparison cohort consisted of 21, 574 matched control patients without hyperthyroidism. The incidence of bipolar disorders (incidence rate ratio [IRR], 2.31, 95% CI 1.80–2.99, *P*<.001) was higher for the hyperthyroidism patients than the control patients. Multivariate, matched regression models showed that women (HR 2.02, 95% CI 1.34–3.05, *P* = .001), patients with alcohol use disorders (HR 3.03, 95% CI 1.58–5.79, *P* = .001), and those with asthma (HR 1.70, 95% CI 1.18–2.43, *P* = .004) were independent risk factors for the development of bipolar disorders in hyperthyroidism patients.

**Conclusions:**

Although a possibility that the diagnosis of bipolar disorders in this study actually includes "bipolar disorders due to hyperthyroidism" cannot be excluded, this study suggests that hyperthyroidism may increase the risk of developing bipolar disorders.

## Introduction

With recent advances in psychoneuroendocrinology, there has been growing interest in the psychological aspects of clinical care in endocrine disease. Thyroid disorders have long been associated with psychiatric illness, often with symptoms suggestive of mood disorders. The most common clinical features associated with hyperthyroidism have been anxiety and depression. [1] Bipolar disorders, especially bipolar mania, is less commonly documented [2].

Studies have shown that mood disturbance often accompanies hyperthyroidism in patients. [3] Substantial numbers of patients with hyperthyroidism exhibit features of depressive disorders, [4] and some patients with hyperthyroidism develop mania-like symptoms. [5] Such patients can be diagnosed with mood disorders due to hyperthyroidism because the symptoms of depressive and bipolar disorders are the direct physiological effects of hyperthyroidism, according to the criteria of the Diagnostic and Statistical Manual of Mental Disorders, Fourth Edition (DSM-IV). However, the mechanisms underlying such direct effects are unclear.

Prospective studies investigating the effect of successful treatment of hyperthyroidism on mood disturbance showed that both mood disturbance and hyperthyroid symptoms subsided equally with the treatment. [4] However, other studies have shown that symptoms of bipolar disorders persist in patients with hyperthyroidism after their hyperthyroidism has been brought under control with medication. [6], [7] Moreover, another study stressed that residual mood disturbance may exist for a long time even under euthyroid status. [8] There is no doubt that elevated thyroid hormone level has direct effect on mood. However, we can also hypothesize that a history of hyperthyroidism increases the risk of subsequent onset of "idiopathic" bipolar disorder.

To prove our hypothesis, we designed a nationwide population-based study to investigate the actual incidence of bipolar disorders in patients with hyperthyroidism in Taiwan.

## Patients and Methods

### Data Sources

The Taiwan National Health Insurance (NHI) program, offers a comprehensive, unified universal health insurance program to all residents of Taiwan. The NHI program covers more than 96% of the residents of Taiwan, and has contracted with 99% of all the hospitals and clinics in Taiwan. [9] The program includes coverage for outpatient, inpatient, emergency, and traditional Chinese medicine services, as well as for prescription drugs. Multiple NHI databases, including NHI enrolment files, claims data, and a prescription drug registry, are managed and publicly released by the National Health Research Institute (NHRI) of Taiwan. The Bureau of NHI and the NHRI regulations guarantee patient confidentiality, and data that may have identified a patient were encrypted, therefore, written consent from study objects was not obtained and this study was exempt from full review by the Institutional Review Board of Taipei Veterans General Hospital, as the NHI dataset consists of de-identified secondary data for research purposes.

### Study Design and Participants

We conducted a retrospective cohort study of patients who were newly diagnosed with hyperthyroidism between January 1, 2000, and December 31, 2010. We identified hyperthyroidism cases in the Taiwan National Health Insurance Research Database (NHIRD) based on the International Classification of Diseases, 9th revision, Clinical Modification (ICD-9-CM) codes 242 (thyrotoxicosis with or without goiter). To increase the validity of hyperthyroidism diagnoses, we included only the patients who received laboratory analysis of serum level of thyroid-stimulating hormone and thyroxine and diagnosed as hyperthyroidism. Patients with bipolar disorders were identified based on the diagnosis of a mood or behavior disturbance related to the principal diagnosis of bipolar disorders (ICD-9-CM code 296.0X, 296.1X, 296.4X, 296.5X, 296.6X, 296.7X, 296.80, or 296.89) between January 1, 2000, and December 31, 2010. We also collected information on the use of mood stabilizer drugs, and the mood stabilizer drugs were classified according to the World Health Organization (WHO) Anatomical Therapeutic Chemical (ATC) classification. Only patients who received a mood stabilizer prescription for at least one month were included in our study. In addition, patients with mood disorders due to a general medical condition (ICD-9-CM code 293.83) were not included and patients with a history of mood disorders before the enrollment date were excluded from our study.

For each patient with hyperthyroidism in the NHIRD, one patient without hyperthyroidism matched for age, sex, comorbidities, [10] and enrollment date was selected. The same exclusion criteria were applied to the matched comparison cohort. Both the hyperthyroidism and comparison patients were followed until the development of bipolar disorder, death, or the end of the study period.

### Statistical Analysis

Diagnosis of bipolar disorders served as the primary dependent variable. We calculated the bipolar disorder incidence rates (per 1000 person-years) and the incidence rate ratios (IRRs). Comparisons between the study groups were made using the χ^2^ test for categorical variables. The Kaplan-Meier method was used to estimate the cumulative incidence of bipolar disorders, and a Cox proportional hazards model was used to identify risk factors for bipolar disorders in patients with hyperthyroidism. The qualifying criterion for inclusion in the multivariate analysis was a result in the univariate-analysis with a *P* value of less than 0.1. Because of a potential ascertainment bias, we tried to examine two diagnoses of the other neuropsychiatric disorder which is not assumed to be related to thyroid function, as “disease control”. The first is intracranial hemorrhage (ICH) and the other is malignant neoplasm of brain. The Perl programming language (version 5.12.2) was used to extract the data from the databases. Microsoft SQL Server 2005 (Microsoft Corp., Redmond, WA, USA) was used for data linkage, processing, and control sampling. The SPSS, version 19.0 for Windows (IBM, Armonk, NY, USA), and the SAS, version 9.2 (SAS Institute, Cary, NC, USA), computer software programs were used to perform all the statistical analyses. Results of comparisons with a *P* value less than.05 were considered statistically significant.

## Results

### Participant Characteristics

Table 1 showed the demographic and comorbidity data for the hyperthyroidism patients and control participants. The median age of the patients was 41 years (interquartile range, 31 to 52 years). The majority of patients in both cohorts were women (77.6%). Hypertension, diabetes mellitus, chronic obstructive pulmonary disease, and asthma were the most common comorbidities. There were no statistically significant differences in the baseline comorbidity data between the study groups.

**Table 1:** Baseline characteristics of patients with and without hyperthyroidism.

Demographic data	Patients with Hyperthyroidism*N = *21, 574			Patients without Hyperthyroidism*N = *21, 574		*P* value
	*n*	percent	*n*		percent	
**Age (y) (Interquartile range)**	41(31–52)		41(31–52)			
≥40	11323	52.5	11323		52.5	1.000
<40	10251	47.5	10251		47.5	
**Sex**						
Men	4,827	22.4	4,827		22.4	1.000
Women	16,747	77.6	16,747		77.6	
**Comorbidities**						
Alcohol use disorders	509	2.4	503		2.3	.849
Autoimmune diseases	1761	8.2	1750		8.1	.846
Chronic kidney disease	1,928	8.9	1,922		8.9	.919
Cerebrovascular disease	1,765	8.2	1,752		8.1	.819
Diabetes mellitus	3,857	17.9	3,851		17.9	.940
Drug abuse	79	0.4	59		0.3	.088
Hypertension	4,948	22.9	4,954		23.0	.945
Coronary artery disease	193	0.9	170		0.8	.225
Asthma	2,567	11.9	2,569		11.9	.976
COPD	3,571	16.6	3,565		16.5	.938
**Follow-up years (median)**	5.98(3.42–8.53)		5.95(3.41–8.51)			.634

COPD, chronic obstructive pulmonary disease.

### Incidence of Bipolar Disorder

**Figure 1:**
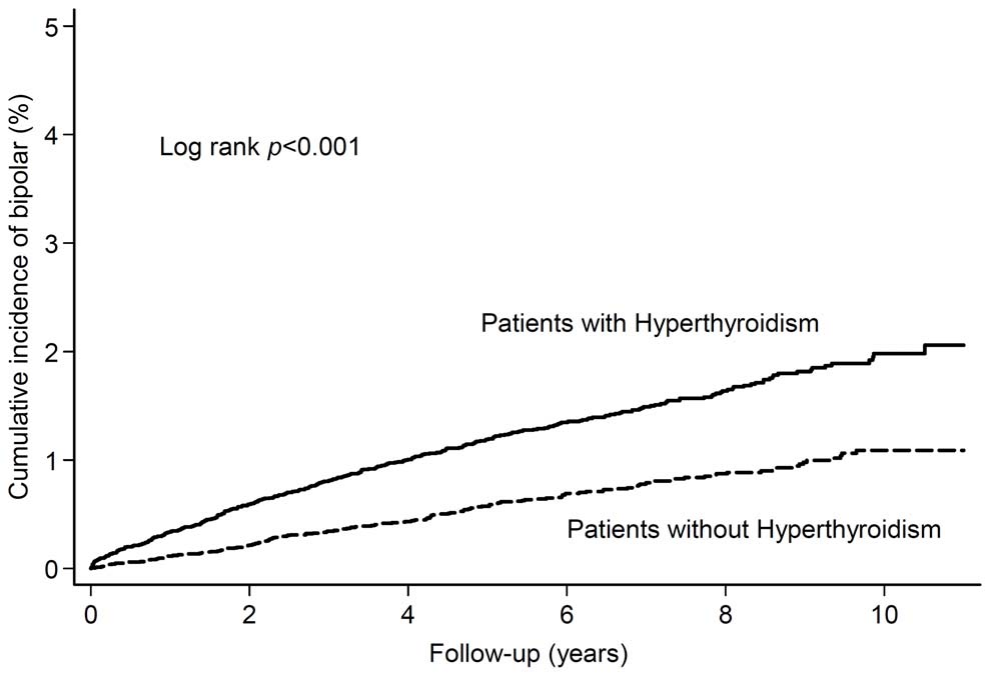
Cumulative incidence of subsequent bipolar disorders in patients with and without Hyperthyroidism.

The cumulative incidences of bipolar disorders were shown in Figure 1. As shown in Table 2, the risk of developing bipolar disorders risk was significantly higher for patients with hyperthyroidism than the matched control patients (IRR, 2.31; 95% CI, 1.80–2.99; *P*<.001). The stratification of patients by age, sex, and follow-up duration had similar risk of developing bipolar disorders in either cohort. Overall, our study showed that the incidence of the development of bipolar disorders after the diagnosis of hyperthyroidism was 1.6 per 1000 person-years.

**Table 2:** Incidence of bipolar among patients with and without hyperthyroidism.

		Patients with hyperthyroidism		Patients without hyperthyroidism		Risk ratio (95% CI)	*P* value
		Bipolar	Per 1,000 person-years	Bipolar	Per 1,000 person-years		
Total	211		1.6	91	0.7	2.31(1.80–2.99)	<.001
Age							
≥40 y	101		1.6	45	0.7	2.25(1.57–3.27)	<.001
<40 y	110		1.7	46	0.7	2.38(1.67–3.44)	<.001
Sex							
Men	27		1.0	12	0.4	2.24(1.10–4.85)	.017
Women	184		1.8	79	0.8	2.33(1.78–3.07)	<.001
Follow-up							
0–0.5 y	30		2.8	9	0.8	3.34(1.55–8.00)	<.001
0.5–1 y	20		1.9	8	0.7	2.50(1.06–6.57)	.024
1–5 y	121		1.8	53	0.8	2.28(1.64–3.22)	<.001
≥5 y	40		1.0	21	0.5	1.89(1.09–3.37)	.017

CI, confidence interval.

### Risks Factors for Bipolar Disorder in Patients with Hyperthyroidism

In the univariate and multivariate analyses, female sex, alcohol use disorders, and asthma were independent risk factors for the development of bipolar disorders in hyperthyroidism patients (Tables 3).

**Table 3:** Analyses of risk factors for bipolar in patients with hyperthyroidism.

Predictive variables	Univariate analysis		Multivariable analysis	
	HR (95% CI)	*P* value	HR (95% CI)	*P* value
Age ≥40 y	0.89 (0.68–1.16)	.381		
Sex (female)	1.85 (1.24–2.77)	.003	2.02 (1.34–3.05)	.001
Comorbidities				
Alcohol use disorder	3.12 (1.74–5.58)	<.001	3.03 (1.58–5.79)	.001
Autoimmune diseases	1.58 (1.02–2.43)	.040		
Chronic kidney disease	1.18 (0.74–1.87)	.486		
Cerebrovascular disease	1.65 (1.08–2.52)	.021	1.54 (1.00–2.36)	.049
Diabetes mellitus	0.96 (0.67–1.39)	.842		
Drug abuse	5.72 (2.13–15.39)	.001	2.86 (0.97–8.46)	.058
Hypertension	1.23 (0.90–1.68)	.199		
Coronary artery disease	1.20 (0.30–4.83)	.798		
Asthma	1.82 (1.28–2.60)	.001	1.70 (1.18–2.43)	.004
COPD	1.34 (0.95–1.89)	.100		

COPD, chronic obstructive pulmonary disease.

### The Analysis of Disease Control

In the group of ICH, 212 incident cases developed in patients with hyperthyroidism, and 199 in matched cohort. Incident rate is 1.38 per 1,000 person-years and 1.30 per 1,000 person-years. *P* value = 0.564. In the group of malignant neoplasm of brain, 7 incident cases developed in patients with hyperthyroidism, and 7 in matched cohort. Incident rate is 4.61 per 100,000 person-years and 4.61 per 100,000 person-years, respectively. *P* value = 1.

## Discussion

This is the first population-based study to examine hyperthyroidism as a risk factor for bipolar disorders using a matched cohort study and a long-term (11-year) follow-up period. The major finding of our study is the higher incidence of subsequent bipolar disorders in patients with hyperthyroidism. In addition, women with hyperthyroidism had a greater risk of developing bipolar disorders than men with hyperthyroidism. Furthermore, alcohol use disorders and asthma may be risk factors for the development of bipolar disorders in patients with hyperthyroidism.

In Denmark, a large-scale inpatient registry-based study investigated the relationship between hyperthyroidism and mood disorders, [11] and found that among 28 190 patients with hyperthyroidism, the incidence of subsequent bipolar disorders was higher (0.17%) than that of the control patients during a 22-year observational period. Although our results are in agreement with such findings, our study found that the increase in the incidence of subsequent bipolar disorders in our hyperthyroidism patients, compared with control patients, was much higher (0.98%) during the 11-year observational period of our study than the increase seen in the Danish cohort. There are some reasons which could explain these two discrete findings. First, referral bias could not be avoided in the registry-based study design; Second, inpatient study design could not obtain data regarding patients with hyperthyroidism and bipolar disorders who were treated in outpatient settings.

In our study, patients with hyperthyroidism were at higher risk for subsequent bipolar disorders. We hypothesize that it may be linked to two possible mechanisms. First, bipolar disorders development after hyperthyroidism may be seen as a result of inflammatory process. Studies have shown that the activation of the inflammatory process may underpin the pathophysiology of many chronic inflammatory diseases, including hyperthyroidism and bipolar disorders.[11]–[13] Second, residual emotional deficits following hyperthyroidism may also make patients more prone to developing bipolar disorders [8].

In epidemiological studies, although gender differences in age of onset in bipolar I disorder were well found, there is no conclusive proof that women had higher risk of developing bipolar disorders than men. [14] Moreover, some studies revealed that the onset of bipolar disorder is not associated with gender. [15] However, women with hyperthyroidism were at greater risk of developing bipolar disorders than the men in our study. It is possible that mechanisms associated with thyroid hormones and estrogens may have overlapping functions. [16] Studies has shown that women who are vulnerable to fluctuating estrogen levels may be at increased risk for developing bipolar disorders [17].

In our analysis of the risk factors associated with subsequent bipolar disorders in hyperthyroidism patients, alcohol use disorders were an independent risk. Evidence has shown that alcohol use disorders and bipolar disorders share certain common genetic characteristics, neuroimaging findings, and biochemical findings [18], [19].

We also found that hyperthyroidism patients with a history of asthma were at greater risk for developing bipolar disorders. Asthma is a common comorbidity in hyperthyroidism patients with bipolar disorders, [12] and previous studies have shown that asthma is associated with a significantly greater likelihood of developing bipolar disorders. [20] It is now well accepted that asthma is an inflammatory disease of the airways [21] and therefore one possible explanation of this association is that chronic inflammation contributes to both hyperthyroidism and bipolar disorders.

In the analysis of disease control, the results clearly showed that the observed increased incidence of bipolar disorder is not due to ascertainment bias.

Our study is one of the few retrospective studies that have examined hyperthyroidism as a risk factor for the development of bipolar disorders using a population-based cohort of patients in Taiwan. A matched case-controlled study design using a cohort of hyperthyroidism patients and adequate controls for comorbidities constitute the strengths of our study. However, several limitations that are inherent to the use of claims databases should be considered. First, the diagnosis of hyperthyroidism in the NHIRD was based on the ICD-9 code for hyperthyroidism and the laboratory survey arrangement. The serum level of thyroid hormone could not be obtained from the database. Under this limitation, whether the severity of hyperthyroidism is risk factor for subsequent bipolar disorders is difficult to explore. Second, in our study, we included only those patients who had been diagnosed with hyperthyroidism and later diagnosed as bipolar disorder. The differential diagnosis between idiopathic bipolar disorder and bipolar disorder due to hyperthyroidism was solely based on clinical judgment by the attendant physician. Therefore, having “idiopathic bipolar disorder” diagnoses cannot rule out a possibility that the patients actually have “bipolar disorders due to hyperthyroidism”. Moreover, causal relationship was assessed mainly by the time order of these two conditions. However, both conditions may take a long time to come to the treatment and thus a possibility that bipolar disorders caused hyperthyroidism cannot be totally excluded from these results. Lastly, no information regarding family history and smoking status were available in the NHIRD either, both of which may have provided useful information regarding factors that may be associated with thyroid abnormalities or bipolar disorders [22], [23].

In conclusion, although a possibility that the diagnosis of bipolar disorders in this study actually includes “bipolar disorders due to hyperthyroidism” cannot be excluded, this study suggests that hyperthyroidism may increase the risk of developing bipolar disorders. Based on our data, we suggest that more attention should be focused on female patients, patients with alcohol use disorders, and those with asthma following a diagnosis of hyperthyroidism. Further prospective clinical studies of the relationship between hyperthyroidism and bipolar disorders are warranted.
